# Excellent Characteristics of Environmentally Friendly 3D-Printed Nasopharyngeal Swabs for Medical Sample Collection

**DOI:** 10.3390/polym15163363

**Published:** 2023-08-10

**Authors:** Ahmad Mamba’udin, Murni Handayani, Farid Triawan, Yosephin Dewiani Rahmayanti, Muhammad Akhsin Muflikhun

**Affiliations:** 1 Mechanical and Industrial Engineering Department, Gadjah Mada University, Jl. Grafika No.2, Yogyakarta 55281, Indonesia; 2 Research Center for Advanced Materials, National Research and Innovation Agency (BRIN), Puspiptek Area, Tangerang Selatan 15314, Indonesia; 3 Department of Mechanical Engineering, Sampoerna University, Jl. Raya Pasar Minggu No.Kav. 16, Kec. Pancoran, Jakarta 12780, Indonesia

**Keywords:** nasopharyngeal swab, vat photopolymerization, additive manufacturing, COVID-19

## Abstract

3D-printed nasopharyngeal swabs for medical sample collection have been manufactured via additive manufacturing (AM), evaluated, and characterized in the present study. A multi-part component of nasopharyngeal swabs was proposed, in which the swab and handle were manufactured separately to reach sustainable production and environmentally friendly products. The swab was investigated using tensile, flexural, surface roughness, dimensional accuracy, and sample collection testing. The influence of printing parameters and post-curing time treatment on the mechanical properties, surface roughness, and dimensional accuracy of 3D-printed nasopharyngeal swabs were also evaluated. The result showed that 3D-printed nasopharyngeal swab shows outstanding tensile strength compared to the commercial flock nasopharyngeal swab. Moreover, the swab neck flexibility test showed that both PLA and dental non-castable 3D-printed nasopharyngeal swabs were able to bend 180°. Subsequently, the surface roughness of 3D-printed nasopharyngeal swab was identic with the commercial flock nasopharyngeal swab. The proposed 3D-printed nasopharyngeal swab design could carry an artificial mucus sample of 141.6 mg at a viscosity of 9455.4 mPa.s. The cost to fabricate a 3D-printed nasopharyngeal swab was estimated at USD0.01–0.02 per swab. 3D-printed nasopharyngeal swab shows potential as a feasible option, greener, less medical waste, and more sustainable.

## 1. Introduction

Coronavirus disease (COVID-19) mutations still occur in many countries [1]. Concerns about the presence of new omicron subvariants take more attention from various parties [2,3]. Since the beginning of the COVID-19 pandemic, three-dimensional printing (3DP) has become an important technology in the development of medical device design [4] and supports enhanced healthcare and emergency response [5]. 3DP technology, also known as additive manufacturing (AM), creates three-dimensional objects layer-by-layer or point-by-point in a subsequent manner from a digital 3D model [6,7]. 3DP technologies have been popularly applied in different applications from medical devices, art, oil and gas industries, and outdoor equipment [8,9,10,11,12,13,14,15] due to advances in the development of printable materials [7] and the ability of 3DP technologies to produce complex structures on a micro-scale [16]. Furthermore, 3DP technology provides faster, easier, minimum waste, reduced energy, and inexpensive solutions for various applications [12,13,15]. According to ISO/ASTM 52900:2021, AM processes are classified into seven categories based on the basic principle of their operation: binder jetting (BJ), directed energy deposition (DED), material extrusion (ME), material jetting (MJ), powder bed fusion (PBF), sheet lamination (SL), and vat photopolymerization (VP). VP has the best print quality compared to other AM methods in terms of printing resolution [17], surface roughness [18], dimensional accuracy [19], and low porosity of printed parts [20] for basic shapes, but a limited choice of materials as the photopolymer resin material has to be photopolymerizable [21]. VP changes the liquid material, in this case, photopolymer resin, into a solid material layer by layer using light in the photopolymerization process [14]. VP can be categorized into three types based on the mechanism of light exposure to the resin liquid: stereolithography (SLA), digital light processing (DLP), and liquid crystal display (LCD) printing [22].

Recently, the spread of COVID-19 still concerns scientists because the COVID-19 virus continues to mutate. COVID-19 testing is crucial to control the deployment of COVID-19 because it functions to find patients or individuals affected by COVID-19 to immediately isolate or limit close contact with the people around them [23]. One way to find out patients infected by the COVID-19 virus is by carrying out a PCR swab test (polymerase chain reaction), where ribonucleic acid (RNA) is detected using nucleic acid with the help of a PCR reaction, which is sampling respiratory was obtained using a nasopharyngeal swab [24]. A nasopharyngeal swab is inserted through the nostril until it reaches the inferior turbinate and back of the nasopharynx. The Nasopharyngeal swab was then rotated several times and pulled out. After sampling, a nasopharyngeal swab was put into a vial containing several milliliters of liquid solution for further virus check [25].

At the beginning of the COVID-19 pandemic, many countries faced shortages of nasopharyngeal swabs due to increased demand and decreased supply for COVID-19 testing [26]. 3DP technology has emerged to solve the shortages as an alternative manufacturing method and design development of nasopharyngeal swabs [25]. 3DP technologies have several advantages for the rapid development of nasopharyngeal swabs, such as fast prototyping for iteration design, the multiplicity of material, and printing processes available [22], which means there are many alternatives to create a swab and 3D printers already used in hospitals and clinic so that they can print their swabs to fulfill the demand [23]. In addition, the ability to transmit the nasopharyngeal swab models using various platforms and the internet and then print them in a particular place means that the manufacture of nasopharyngeal swabs using 3DP technology can overcome the supply chain disruption that will occur and simultaneously reach all user [27].

Many studies have been conducted on the development of 3D-printed nasopharyngeal swabs. Alghounaim et al. [28] compared 3D-printed nasopharyngeal swabs to commercially manufactured swabs. Polylactic acid and polyester were chosen as source materials because they were inexpensive and easy to get. Fused deposition modeling (FDM) 3D printer was used to manufacture the prototype of a nasopharyngeal swab. They reported that polylactic acid and polyester 3D-printed nasopharyngeal swabs were effective for COVID-19 sample collection. Williams et al. [29] developed a 3D-printed nasopharyngeal swab using a selective laser sintering (SLS) printer and medical-grade bio-compatible nylon as source material. Design optimization was developed on the tip geometry swab for maximum sample collection, shaft geometry swab for flexibility, easy to use, and ensured overall design parameters were suitable for patient comfort. Ford et al. [30] developed and manufactured a 3D-printed nasopharyngeal swab via a stereolithography (SLA) 3D printer since it was easily accessible for biocompatible and inexpensive materials. Tay et al. [31] designed and clinically validated a 3D-printed nasopharyngeal swab. The swab was printed from medical grade resin and broke at an average load of 40 N, also able to carry fluid of 38 µL. A novel design was developed for realizing patient-specific nasopharyngeal swabs using MATLAB and the patient’s CT data [32]. Furthermore, a creative design of 3D-printed nasopharyngeal swab for children and infants have been developed by Alazemi et al. [33]. A total of 160 swab designs from various parties have been evaluated and validated four designs by Callahan et al. [25]. In addition to mechanical strength considerations, the nasopharyngeal swab designs have been widely developed to obtain sufficient samples for COVID-19 testing [27,30].

Based on previous studies, vat photopolymerization 3D printing is broadly used for the fabrication of 3D-printed nasopharyngeal swabs because it can produce a superior quality printed part, easy to get in the market, available in a diverse range of materials and relatively more affordable prices compared to other AM methods. Furthermore, materials used in fabricating 3D-printed nasopharyngeal swab using vat photopolymerization still focuses on surgical-grade resins, which is relatively expensive. There have been many studies regarding 3D-printed nasopharyngeal swab fabrication using vat photopolymerization 3D printing. However, no research yet examines the influence of printing parameters and curing process treatment on the characteristics of the 3D-printed nasopharyngeal swab to ensure that the 3D-printed nasopharyngeal swab is suitable for use before being tested clinically. Therefore, this study aims to design, manufacture, and evaluate 3D-printed nasopharyngeal swabs for COVID-19 sample collection with two sections to reduce medical waste and keep the environment cleaner. The influence of printing parameters and curing time treatment on mechanical properties, dimensional accuracy, and surface roughness of 3D-printed nasopharyngeal swabs will be studied. The characteristics and performance of 3D-printed nasopharyngeal swabs were evaluated. A novel 3D-printed nasopharyngeal swab consisting of a multi-part component was proposed. 3D-printed nasopharyngeal swabs were subjected to mechanical tensile testing, surface roughness testing, dimensional accuracy testing, and sample collection testing for preclinical testing. A digital light processing (DLP) 3D printer machine was used to manufacture 3D-printed nasopharyngeal swabs using PLA PRO photopolymer resin and dental non-castable photopolymer resin as source material. The defectiveness of the 3D-printed nasopharyngeal swab manufacturing process was also presented.

## 2. Materials and Methods

### 2.1. Nasopharyngeal Swab Design

**Figure 1:**
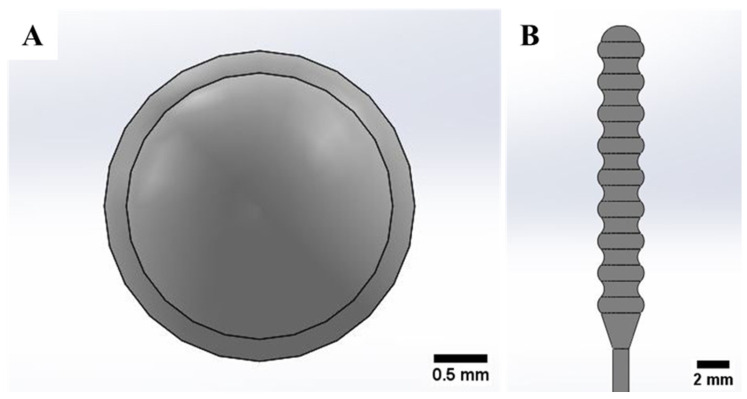
Swab head. (**A**) Swab head tip, (**B**) side view of swab head [34].

The nasopharyngeal swab should be able to carry a sufficient virus sample in the form of the patient’s mucus from the nasopharynx, retain, and release the patient’s mucus to a transport vial for detection of viral nucleic acid. Nasopharyngeal swabs also should have a smooth surface for patient comfort and safety. No less important, the nasopharyngeal swab must be flexible enough while moving into the posterior nasopharynx. Based on these considerations, the swab head tip is designed to be rounded to minimize pain or discomfort experienced by patients when a nasopharyngeal swab is used, as seen in Figure 1A. The swab head has peaks and valleys sections (see Figure 1B) to ensure the collection and retention of sufficient patient mucus samples during the testing. The rear of the swab head is narrowed for easy penetration of the swab.

**Figure 2:**
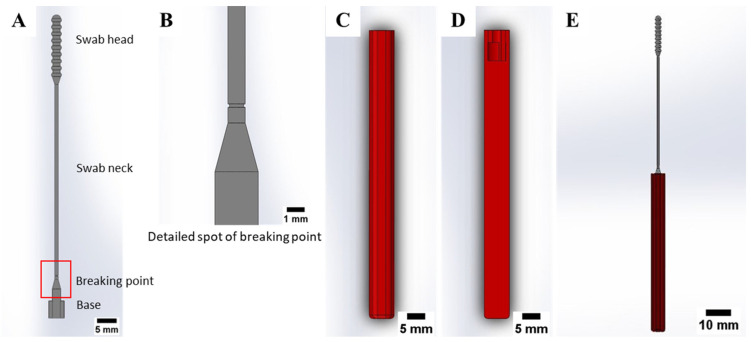
3D-printed nasopharyngeal swab model. (**A**) Full side view swab, (**B**) Detailed spot of breaking point, (**C**) Handle, (**D**) Section view of handle, (**E**) Swab and handle assembled [34].

The nasopharyngeal swab consisting of a multi-part component was proposed, as shown in Figure 2, so that the swab and swab handle can be manufactured separately, and the swab size becomes shorter. This gives several of the following benefits: (i) reducing the printing time, (ii) reducing the amount of photopolymer resin material, (iii) allowing the nasopharyngeal swab to be printed on smaller print volumes VP printer, (iv) speeding up the printing process of the swab handle on the other VP 3D printer which the swab handle can be used more than once testing after sterilization process. The swab handle printing process can be accelerated because the swab handle was printed in a planar alignment.

Based on the tensile test, the standard flock nasopharyngeal swab showed that the swab was frequently broken in the middle section of the swab neck when subjected to mechanical loading. This can create a risk for the patient because the nasopharyngeal swab can be broken in the patient’s nasal cavity during testing. To ensure the nasopharyngeal swab is broken at the exact location outside the patient’s nasal cavity, nasopharyngeal swabs are designed to fail or break at a particular position. The nasopharyngeal swab was given the stress concentration or breaking point located at the bottom section of the swab neck in the form of a reduction in the size of the neck diameter to 0.8 mm, as shown in Figure 2A,B. At this point, the swab has the smallest cross-sectional area. Thus, it has the weakest strength of the other parts. The handle and swab are used plug-and-play, with an available locking mechanism between the swab and the handle. The swab locking mechanism is done by rotating the swab position by 90° after the swab is inserted into the handle (see Figure 2C,D). The assembled swab can be seen in Figure 2E. The final dimensions of the swab are as follows: diameter base is 2.5 mm, neck length is 57 mm with a diameter of 1 mm, swab head length is 21 mm with a diameter of 2.9 mm, and total length is 89 mm. The swab handle has a length of 80 mm with a thickness of 6 mm [34].

### 2.2. Materials

Material selection was considered by three standards: (i) mechanical ability, (ii) availability in the market, and (iii) affordability. Based on our discussion, the material should be less expensive and easily adopted worldwide. PLA PRO photopolymer resin by eSun (Shenzhen, China) and dental non-castable photopolymer resin by Anycubic (Shenzhen, China) was selected as source materials to manufacture 3D-printed nasopharyngeal swab. In addition, the use of PLA PRO photopolymer resin and dental non-castable photopolymer resin as source material for 3D-printed nasopharyngeal swabs is still not widely explored. The swab handle was manufactured using standard photopolymer resin by eSun (Shenzhen, China). The chemical compositions and properties of the photopolymer resin are shown in Table 1 and Table 2.

### 2.3. 3D Printing and Specimen Preparation

**Figure 3:**
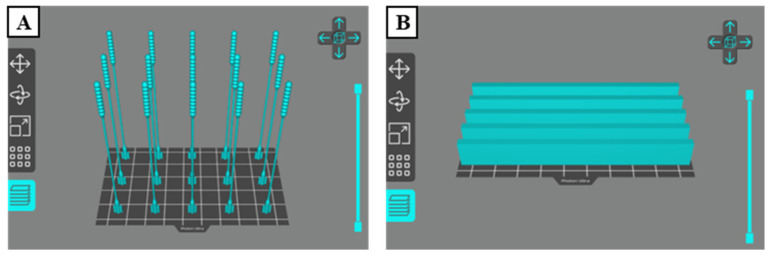
Build orientation specimen on software slicer. (**A**) 3D-printed nasopharyngeal swab, (**B**) Flexural test specimen.

There are two types of test specimens in the present study: 3D-printed nasopharyngeal swabs and flexural test specimens. 3D-printed nasopharyngeal swabs were subjected to tensile testing, surface roughness testing, dimensional accuracy testing, and sample collection testing. In contrast, the flexural test specimens were subjected to flexural testing. The 3D model specimen was made using Solidworks 2019 (United States). The designed model was then saved in the form of STL file. Photon Workshop V2.1.29 (Shenzhen, China) software slicer was used to import the STL file on the build plate and then set the number of specimens, printing orientation, and printing parameter settings. The 3D model specimens were arranged according to the amount to be printed. A total of 15 nasopharyngeal swabs were printed per batch, as shown in Figure 3A, which takes an estimated 3.178 mL photopolymer resin to print. In contrast, the flexural test specimens were printed with five specimens per batch, as shown in Figure 3B, which takes an estimated 20.198 mL photopolymer resin. All specimens were printed with a 90° build orientation, which allows the swabs to be printed in large quantities per batch and reduces the possibility of the swab being damaged. The printing parameters such as layer thickness, normal exposure time, off time, bottom exposure time, Z speed, and bottom layer are set according to Table 3, and then the slicing process is performed. PLA PRO specimens were printed with a Z speed of 2 mm/s, while dental non-castable specimens were printed with a Z speed of 1 mm/s. The build plate design was then transported onto a flash memory drive and inserted into a 3D printer machine to start the printing process.

Anycubic Photon Ultra 3D printer (Shenzhen, China) was used to create a 3D-printed nasopharyngeal swabs and flexural test specimens. Anycubic Photon Ultra 3D printer specification is shown in Table 4. Before beginning the printing process, Anycubic Photon Ultra 3D printer must be leveled. After the printing process finished, the printed specimens were gently removed from the printing platform using plastic scraper. All specimens were then subjected to a post-processing treatment: washing and post-curing. The post-processing treatment was performed using the Anycubic Wash and Cure 2.0 machine (Shenzhen, China). The Anycubic Wash and Cure 2.0 machine’s technical specifications are shown in Table 5. The washing process was carried out with a 96% alcohol liquid as a disinfection agent for 5 min and then dried by lying on a tissue for 10 min. The post-curing process was carried out under UV light with a wavelength of 405 nm.

A series of experiments were performed to evaluate the influence of printing parameters and post-curing time on the characteristics of a 3D-printed nasopharyngeal swab. The printing parameter studied is layer thickness. There are three values of layer thickness (LT) will be studied: 0.05, 0.075, and 0.1 mm. In order to evaluate the influence of post-curing time treatment, three different post-curing times (CT) were used as follows: 10, 20, and 30 min. The complete design of the experiments is presented in Table 6.

**Figure 4:**
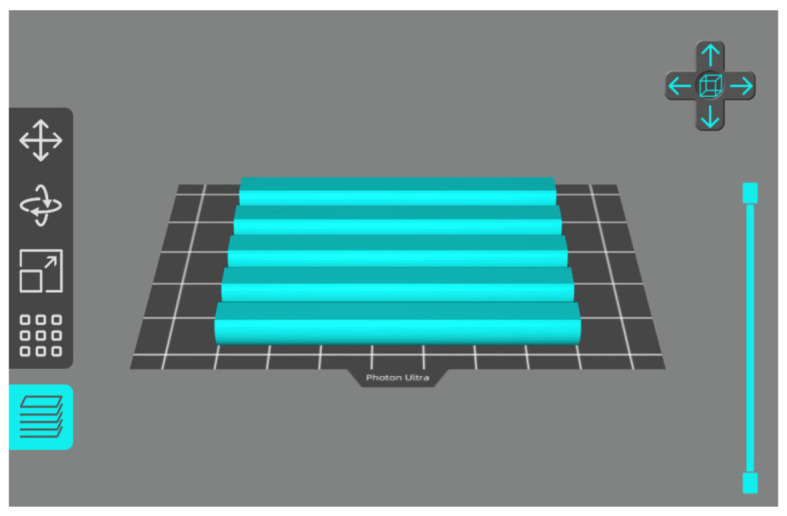
Build plate design of swab handle fabrication.

The swab handle fabrication was printed using printing parameters as follows: layer thickness of 0.05 mm, normal exposure time of 4 s, and bottom exposure time of 40 s. The other printing parameters follow the default setting of the Photon Workshop V2.1.29 software slicer (ET of 3 s, LT of 0.05 mm, Z speed of 2 mm/s). The swab handle can be printed in five pieces per batch on Anycubic Photon Ultra, as shown in Figure 4, which takes 30 min and an estimated 13.577 mL photopolymer resin to print. The swab handle printing parameter is not crucial as long as the swab handle fits well to the nasopharyngeal swab.

3D-printed nasopharyngeal swabs and swab handles need to be sterilized. The sterilization had better be a non-auto-clave method due to the thermal degradation possibility. The sterilization can be done using the STERRAD sterilization method, in which hydrogen peroxide vapor is diffused into a chamber, then inducing the hydrogen peroxide molecules to a plasma state [35]. This method can rapidly sterilize the swabs and handles without toxic residues.

### 2.4. Testing Methods

#### 2.4.1. Sample Collection Test

**Figure 5:**
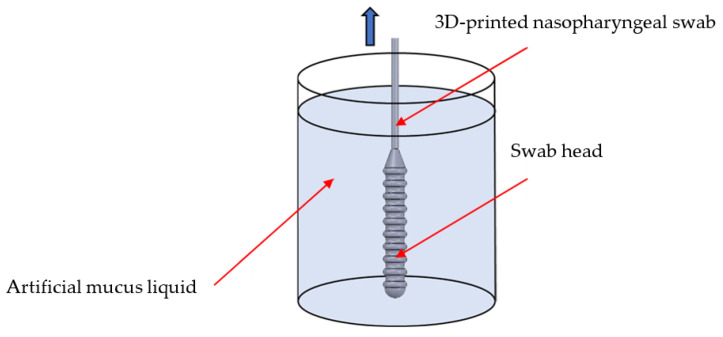
Sample collection testing schematic.

**Figure 6:**
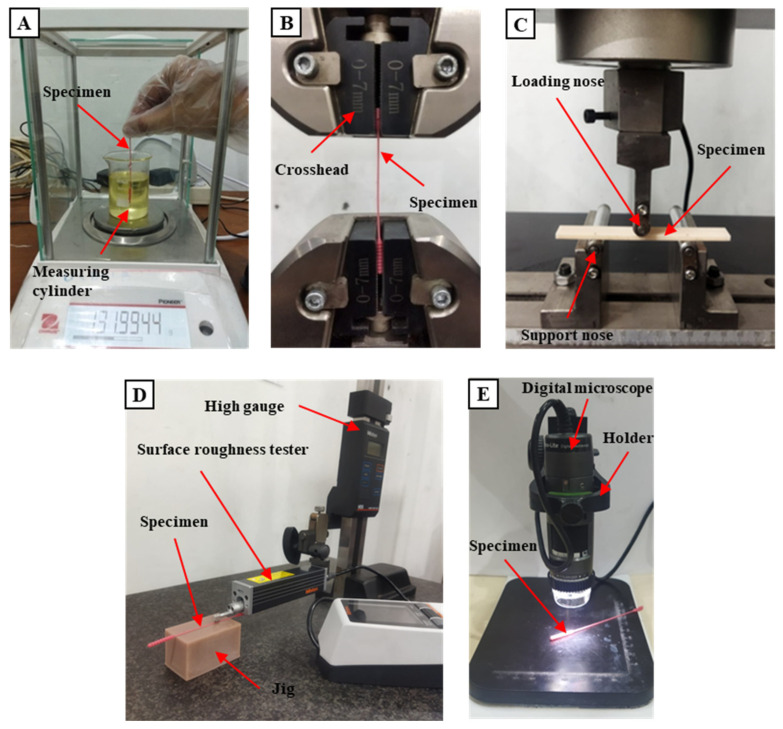
Testing conditions. (**A**) Sample collection test, (**B**) Tensile test, (**C**) Flexural test, (**D**) Surface roughness test, (**E**) Dimensional accuracy test.

One of the criteria that a 3D-printed nasopharyngeal swab must meet is the swab should be able to carry enough samples [25]. A sample collection test was carried out to measure the sample number that can be carried using a 3D-printed nasopharyngeal swab. A sample collection test was performed by dipping a 3D-printed nasopharyngeal swab into the measuring cylinder along the central axis containing artificial mucus until the swab head was completely submerged. A 3D-printed nasopharyngeal swab was then rotated 360° five times, pulled back out from the measuring cylinder, and held for about 20 s until no more artificial mucus liquid dripped into the measuring cylinder. Artificial mucus was prepared by mixing epoxy resin bisphenol A (Eposchon) with acetone to imitate human mucus with different viscosity. A sample collection test was carried out five times across five different concentrations of artificial mucus. The viscosity of artificial mucus was measured using a digital viscometer NDJ-8S (Beijing, China). Viscosity measurements were carried out with a diameter of 50 mm measuring cylinder within a range of 6–30 rpm at 25 °C. The sample collection ability of the 3D-printed nasopharyngeal swab was calculated using the differences between the mass of the artificial mucus in the swab head before and after the 3D-printed nasopharyngeal swab was immersed. The sample collection testing schematic is shown in Figure 5. The mass samples were measured using the Ohauss Analytical Balance (Parsippany, NJ, USA), as shown in Figure 6A.

#### 2.4.2. Tensile Test

A tensile test was performed to imitate the worst-case scenario of a nasopharyngeal swab stuck in an obstacle and pulled out from the nasopharyngeal nasal cavity until it broke. The swab head and swab base are clamped by the crosshead of the universal tensile machine, as shown in Figure 6B. The tensile test was performed using a universal testing machine CRN-50 by Carson Technology Testing Equipment (Taipei, Taiwan) with a crosshead speed of 5 mm/s. The load required when the 3D-printed nasopharyngeal broke was recorded. Five specimens for each variation were tested.

#### 2.4.3. Flexural Test

A flexural test was performed to imitate the scenario of the nasopharyngeal swab bending as it passed through the nasal cavity. It was carried out according to ASTM D790 with a three-point flexural test method to represent the flexural strength photopolymer resin as the source material of a 3D-printed nasopharyngeal swab. The flexural test specimen has a dimension of 100 × 12.6 × 3.2 mm. Universal testing machine CRN-50 using Carson Technology Testing Equipment (Taipei, Taiwan) was used to perform the flexural test. The test was performed with a crosshead speed of 2 mm/s. The specimen was arranged on two support noses with a span length of 53 mm. The flexural testing condition can be seen in Figure 6C. The specimen was then subjected to uniaxially loading in the middle until the specimen failed. The flexural strength was then determined using the following formula:

$$\sigma_{f}=\frac{3PL_{s}}{{2bd}^{2}}$$

and the flexural modulus was determined using the following formula:

$$E_{f}=\frac{{L_{s}}^{3}\;\cdot \;m}{{4bd}^{3}}$$

where *P* is the highest load that can be resisted by the specimen; *L_s_* is support span length; *b* is the width of a specimen; *d* is the thickness of a specimen; and *m* is the slope of the tangent at an elastic region of the load–displacement curve.

#### 2.4.4. Surface Roughness Test

The surface quality of a 3D-printed nasopharyngeal swab is very important because it will affect the patient’s comfort level when the swab is used. The surface roughness test was performed axially on the swab neck surface using a surface roughness tester Mitutoyo SJ-210 (Kanagawa, Japan), as shown in Figure 6D. The calibration and leveling processes were carried out before conducting the measurement. A jig was used to hold and ensure the 3D-printed nasopharyngeal swab did not move during testing. A surface roughness test was carried out on the different sides of the swab neck five times.

#### 2.4.5. Dimensional Accuracy Test

3D-printed nasopharyngeal swabs were measured and compared to the design dimension to assess the printing accuracy of 3D-printed nasopharyngeal swabs; thus, the error percentage is obtained in the form of absolute error (*AE*) and relative error (*RE*). *AE* was calculated using the following formula:

AE=Mv−Ev

and *RE* was calculated using the following formula:

$$RE=\frac{Mv-Ev}{Ev}\times 100\%$$

where *Mv* is the measured value of a 3D-printed nasopharyngeal swab, and *Ev* is the design dimension of a 3D-printed nasopharyngeal swab.

There are four dimensions of a 3D-printed nasopharyngeal swab to be measured: swab neck diameter, swab head diameter, swab head length, and base diameter. The measurement was performed five times for each type of measurement. Dimensional accuracy testing was carried out using Dino-Lite AF4915 (Taipei, Taiwan) after the swab had been subjected to post-processing treatments, as shown in Figure 6E.

## 3. Result and Discussion

### 3.1. Defectiveness in the Manufacturing Process of 3D-Printed Nasopharyngeal Swab

**Figure 7:**
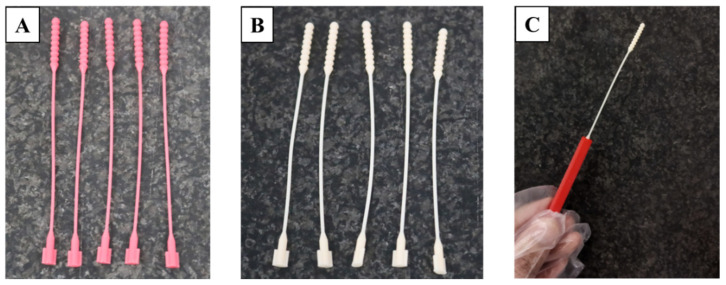
3D-printed nasopharyngeal swab manufacturing results. (**A**) PLA PRO, (**B**) Dental non-castable, (**C**) Assembled swab and handle.

**Figure 8:**
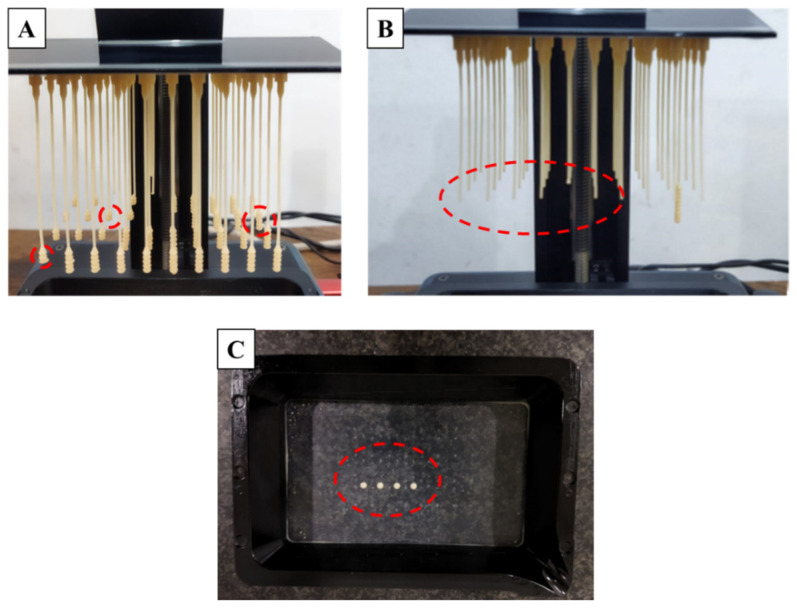
Delamination defect on a 3D-printed nasopharyngeal swab (Z speed of 2 mm/s). (**A**) Delamination on the swab head, (**B**) Delamination on the swab neck, (**C**) The delaminated photopolymer resin attached to the resin vat.

The manufacturing process of nasopharyngeal swabs using vat photopolymerization 3D printing has been carried out in this study. The print results of the 3D-printed nasopharyngeal swab can be seen in Figure 7. There are several manufacturing process defects in the 3D-printed nasopharyngeal swab using a dental non-castable photopolymer resin material. The defect was in the form of a 3D-printed nasopharyngeal swab that could not be completely printed and failed to print on the head and swab neck section, as shown in Figure 8A,B. It is caused by the use of Z speed too high; thus, when the printing platform moves up again, delamination occurs on the swab. Delamination is a print defect where the cured layer of a printed object separates or splits apart from each other. The delaminated photopolymer resin was then attached to the resin vat (Figure 8C), thus obstructing the printing process on the next layer. Delamination also occurs due to less time exposure to UV light. Consequently, the photopolymer resin has not reached sufficient polymerization to be cured.

**Figure 9:**
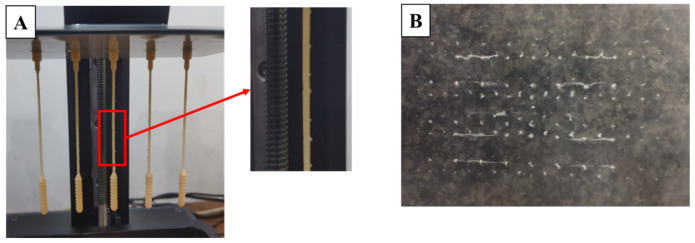
Ragging defect on 3D-printed nasopharyngeal swab (ET 8 s, LT 0.05 mm). (**A**) Ragging on the swab neck, (**B**) Damage in the FEP film resin vat.

**Figure 10:**
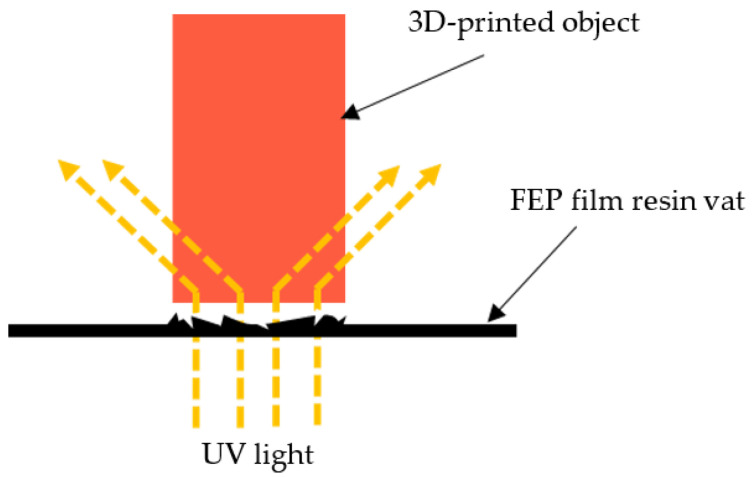
Schematic of UV light scattering.

Another defect in the manufacturing process of the 3D-printed nasopharyngeal swab was the existence of ragging on the swab neck (Figure 9A). Ragging is a print defect where a thin flake of cured or partially cured photopolymer resin extends from the printed object. Ragging might be caused by a scattered UV light that provides sufficient polymerization to the photopolymer resin outside the boundary area of each layer to be cured. The scattering UV light might be caused by any damage in the FEP film resin vat, as shown in Figure 9B. The schematic of UV light scattering during the printing process can be seen in Figure 10. Moreover, the phenomenon of ragging might be related to the low viscosity of dental non-castable resin. After conducting several experiments, a shorter exposure time printing parameter can reduce or even eliminate the presence of ragging on a 3D-printed nasopharyngeal swab. The presence of ragging affects the low surface quality of 3D-printed nasopharyngeal swabs.

### 3.2. Tensile Test

**Figure 11:**
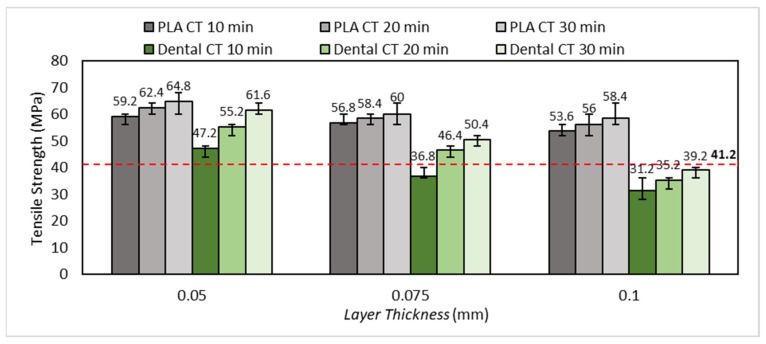
The influence of layer thickness and curing time on the tensile strength of 3D-printed nasopharyngeal swab.

In this section, tensile test results are presented and discussed. The influence of layer thickness and post-curing time on the tensile strength of 3D-printed nasopharyngeal swabs are analyzed. A tensile test was performed on the 3D-printed nasopharyngeal swab with three different values of layer thickness and post-curing time to evaluate the influence of layer thickness and post-curing time on the tensile strength of the 3D-printed nasopharyngeal swab. The commercial flock nasopharyngeal swabs were also tested for comparison. The tensile test result of the 3D-printed nasopharyngeal swab is presented in Figure 11. Based on Figure 11, the highest tensile strength of PLA PRO and dental non-castable 3D-printed nasopharyngeal swabs are 64.8 MPa and 61.6 MPa, respectively, obtained in the printing parameters of LT 0.05 mm at a CT of 30 min. As a comparison, the commercial flock nasopharyngeal swab has a tensile strength of 41.2 MPa (see the red dashed line on Figure 11). The lowest tensile strength of the PLA PRO 3D-printed nasopharyngeal swab is 53.6 MPa, obtained in the printing parameters of LT 0.1 mm at a CT of 10 min. In contrast, the lowest tensile strength of dental non-castable 3D-printed nasopharyngeal swab is 31.2 MPa, which was obtained in the printing parameters of LT 0.1 mm at a CT of 10 min.

The pattern of increasing the tensile strength of PLA PRO and dental non-castable 3D-printed nasopharyngeal swabs shows that the smaller the size of the layer thickness used, the tensile strength value will increase [36,37]. This can be seen in the tensile strength of PLA PRO 3D-printed nasopharyngeal on CT 30 min with an LT of 0.1 mm, 0.075 mm, and 0.05 mm are, respectively, 58.4 MPa, 60 MPa, and 64.8 MPa. Furthermore, The ANOVA result of CT 30 min (with α = 0.05) showed *F*-value (5.20) was higher than its *F*-crit value (3.88). Accordingly, it can be concluded that the tensile strength of the 3D-printed nasopharyngeal swab was influenced by the change in layer thickness. Meanwhile, the ANOVA results of PLA PRO 3D-printed nasopharyngeal swab on CT 20 min and CT 10 min (with α = 0.05) showed *F*-value (8.90) and (10.57), respectively, were also higher than its *F*-crit value (3.88), inferring that there was significant different between the tensile strength of PLA PRO 3D-printed nasopharyngeal swab on CT 20 min and CT 10 min. A similar pattern was also seen for dental non-castable 3D-printed nasopharyngeal swabs, where the obtained tensile strength of 3D-printed nasopharyngeal swabs on CT 30 min with an LT of 0.1 mm, 0.075 mm, and 0.05 mm are 39.2 MPa, 50.4 MPa, and 61.6 MPa, respectively. The ANOVA results on CT 30 min (with α = 0.05) showed *F*-value (147) was far higher than its *F*-crit value (3.88). It can be concluded that there were significant differences between the tensile strength of dental non-castable 3D-printed nasopharyngeal swabs on CT 30 min by the change in layer thickness. Moreover, the ANOVA results of CT 20 min and CT 10 min showed *F*-value (134.57) and (56.18), respectively, were also higher than *F*-crit value (3.88). The tensile strength increases as an increase in the layer numbers, which also causes sufficient curing degree on each layer. A smaller layer size will increase the curing degree. Thus, the adhesion between layers also increases, which leads to an increase in the mechanical properties of printed parts [38].

The obtained tensile test results show (Figure 11) a clear influence of post-curing time on the tensile strength of 3D-printed nasopharyngeal swabs. The tensile strength of PLA PRO 3D-printed nasopharyngeal on LT 0.05 mm with a CT of 10 min, 20 min, and 30 min are 59.2 MPa, 62.4 MPa, and 64.8 MPa, respectively. In addition, the one-way ANOVA result on LT 0.05 mm (with α = 0.05) showed *F*-value (6.16) was higher than its *F*-crit value (3.88), implying that there was a significant difference between the tensile strength of PLA PRO 3D-printed nasopharyngeal swab on LT 0.05 mm by changing the curing time. Otherwise, the tensile strength of PLA PRO 3D-printed nasopharyngeal swab on LT of 0.075 mm and 0.1 mm shows insignificant changes by changing the curing time, which implied in the ANOVA results LT of 0.075 mm and 0.1 mm showed *F*-value (2.40) and (3.37), respectively, were lower than *F*-crit value (3.88). This may be caused by the 3D-printed nasopharyngeal swab being incompletely dried after the washing process, which caused the post-curing process to be suboptimal. Subsequently, the dental non-castable 3D-printed nasopharyngeal swab showed a significant increase in tensile strength at the longest curing time. This can be seen in the tensile strength of 3D-printed nasopharyngeal swabs on LT 0.05 mm with a CT of 10 min, 20 min, and 30 min are 47.2 MPa, 55.2 MPa, and 61.6 MPa, respectively. Additionally, the one-way ANOVA result on LT 0.05 mm (with α = 0.05) showed *F*-value (69.71) was far higher than its *F*-crit value (3.88), which indicates there was a significant difference between the tensile strength of dental non-castable 3D-printed nasopharyngeal swab on LT 0.05 mm by changing the curing time. Furthermore, with α = 0.05, the one-way ANOVA results of LT 0.075 mm and LT 0.1 mm showed *F*-value (57.25) and (13.6), respectively, were also higher than *F*-crit value (3.88). Based on the tensile test results, it can be concluded that increasing CT value affects the tensile strength of 3D-printed nasopharyngeal swabs. These tensile test results are in line with research conducted by Riccio et al. [39], where the post-curing process increases the resistance of the specimen to tensile loads. An increasing in the tensile strength of 3D-printed nasopharyngeal is due to the continuous crosslinking of photopolymer resin under irradiation of UV light [40]. The polymerization degree of crosslinked polymer systems has a vital role in the mechanical properties of materials [41]. The highest tensile strength obtained at CT 30 min showed the 3D-printed nasopharyngeal swab had undergone complete polymerization.

PLA PRO and dental non-castable photopolymer resin materials have different mechanical properties. Based on the tensile test results, the tensile strength of PLA PRO 3D-printed nasopharyngeal swabs was greater than dental non-castable 3D-printed nasopharyngeal swabs. The chemical composition of photopolymer resin affects the mechanical properties of printed specimens [42].

**Figure 12:**
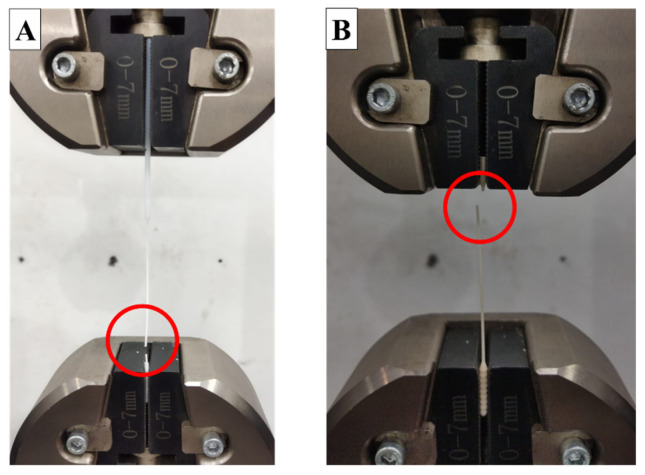
Nasopharyngeal swab fracture location during tensile testing. (**A**) Commercial flock nasopharyngeal swab, (**B**) 3D-printed nasopharyngeal swab.

**Figure 13:**
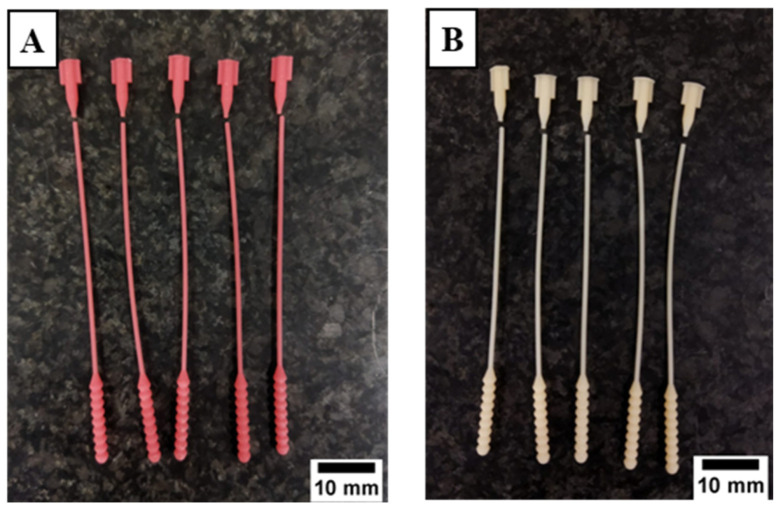
Breaking point evaluation of 3D-printed nasopharyngeal swab (LT 0.05 mm). (**A**) PLA PRO, (**B**) Dental non-castable.

Figure 12 shows the nasopharyngeal swab fracture location when the tensile test was carried out. All 3D-printed nasopharyngeal swab specimens were broken at the breaking point section, as shown in Figure 13. It can ensure the 3D-printed nasopharyngeal swab will not break inside the patient’s nasal cavity. In addition, the commercial flock nasopharyngeal swab was broken in the middle section, as shown in Figure 12A where the 3D printed nasopharyngeal failure at the dedicated breaking point (Figure 12B). It can create a possibility the swab may be broken in the patient’s nasal cavity and cause a new problem in the form of medical action for taking a broken nasopharyngeal swab from the patient’s nasal cavity. From a design point of view, the presence of a breaking point can be a solution alternative to prevent undesirable things from occurring when a nasopharyngeal swab is used in order to ensure a patient’s safety.

### 3.3. Flexural Test

**Figure 14:**
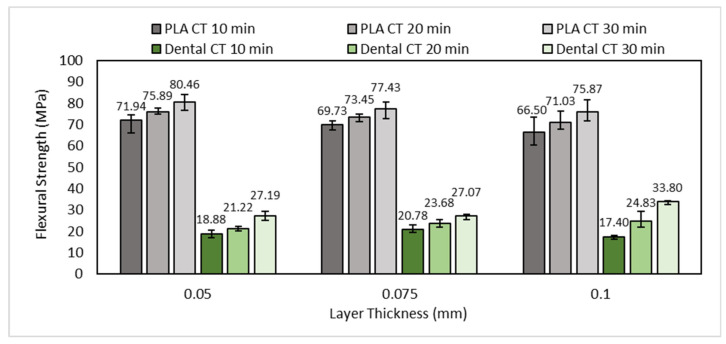
The influence of layer thickness and curing time on the flexural strength specimen.

A flexural test was performed on the flexural test specimen with three different values of layer thickness and post-curing time to evaluate the influence of layer thickness and post-curing time on the flexural strength of the printed part. Based on the flexural test result shown in Figure 14, the highest flexural strength of PLA PRO is 80.46 MPa, obtained in the printing parameters of LT 0.05 mm at a CT of 30 min. In comparison, the highest flexural strength of dental non-castable is 33.80 MPa, obtained in the printing parameters of LT 0.1 mm at a CT of 30 min.

Similar to the tensile test result, the flexural strength of PLA PRO increases with the smaller layer thickness used. As shown in Figure 14, the flexural strength of PLA PRO on CT 20 min with an LT of 0.1 mm, 0.075 mm, and 0.05 mm are 71.03 MPa, 73.45 MPa, and 75.89, respectively. The ANOVA analysis also reveals that there was a significant difference between the flexural strength of PLA PRO on CT 20 min by changing the layer thickness (with α = 0.05, *F*-value (4.85) > *F*-crit (3.88)). The smaller layer thickness will increase the curing degree, which leads to a stronger bond between layers [38]. Thus, it will create greater specimen strength [43]. Otherwise, the influence of layer thickness on dental non-castable shows a random pattern in which the highest flexural strength of dental non-castable obtained on the bigger layer thickness (LT 0.1 mm). However, with α = 0.05, the ANOVA results on CT 10 min (*F*-value (10.47)), 20 min (*F*-value (4.66)), and 30 min (*F*-value (49.21)) showed *F*-value were higher than *F*-crit value (3.88) indicated that there was a significant difference between the flexural strength of dental non-castable by changing the layer thickness.

Based on Figure 14, the influence of curing time on the flexural strength of PLA PRO and dental non-castable is consistent with the study conducted by Nowacki et al. [44], where the flexural strength increases with the longer post-curing time treatment given to the specimen. The flexural strength of PLA PRO on LT 0.05 mm with a CT of 10 min, 20 min, and 30 min are 71.94 MPa, 75.89 MPa, and 80.46 MPa, respectively. This is also proved using the ANOVA result on LT 0.05 mm (with α = 0.05), which showed *F*-value (11.45) was higher than *F*-crit value (3.88), inferring that the flexural strength of PLA PRO was influenced by the change in CT 10 min to 30 min. Moreover, the ANOVA results of LT 0.075 mm and LT 0.1 mm showed *F*-value (14.07) and (5.27), respectively were also higher than *F*-crit value (3.88). A similar pattern is also shown on dental non-castable, which the highest flexural strength obtained on the longer post-curing time. This can be seen in the flexural strength on LT 0.05 mm with a CT of 10 min, 20 min, and 30 min are 18.88 MPa, 21.22 MPa, and 27.19 MPa, respectively. Additionally, the one-way ANOVA result on LT 0.05 mm (with α = 0.05) showed *F*-value (51.07) was higher than its *F*-crit value (3.88), which indicates there was a significant difference between the tensile strength of dental non-castable on LT 0.05 mm by changing the post-curing time. Furthermore, the one-way ANOVA results (with α = 0.05) of LT 0.075 mm and LT 0.1 mm showed *F*-value (27.56) and (115.31), respectively were also far higher than *F*-crit value (3.88). UV light exposure in the post-curing treatment causes continuous crosslinking on the polymer structure [40]. Thus, it will cause the flexural strength of the printed part to increase.

**Figure 15:**
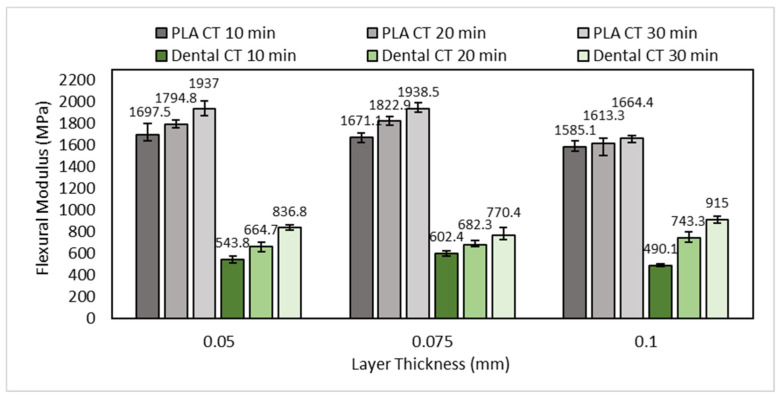
The influence of layer thickness and curing time on the flexural modulus.

The flexural modulus of specimens is presented in Figure 15. Flexural modulus shows the tendency of material stiffness when subjected to a bending load. The higher the flexural modulus of a material, the harder the material being bent. Otherwise, the lower the flexural modulus of a material, the easier the material to be bent. As shown in Figure 15, the flexural modulus of PLA PRO was greater than dental non-castable. This means that PLA PRO is more difficult to bend or, in other words, stiffer than dental non-castable. In general, an increase or decrease in the flexural modulus was linear with the flexural strength. Furthermore, Figure 15 shows the flexural modulus values increase with the longer post-curing time. The longer UV light exposure to the specimen will increase the crosslink density of the polymer structure [45]. The crosslink density causes brittleness of the printed part [46].

**Figure 16:**
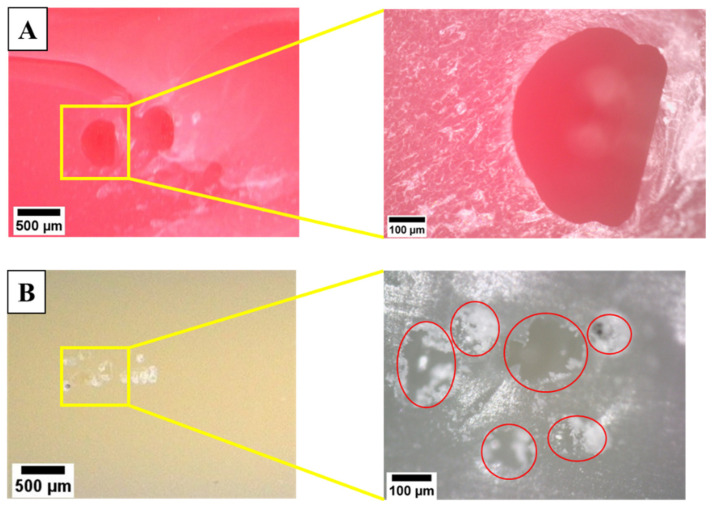
Voids in the fracture surface of the specimen (LT 0.1 mm). (**A**) PLA PRO, (**B**) Dental non-castable.

**Figure 17:**
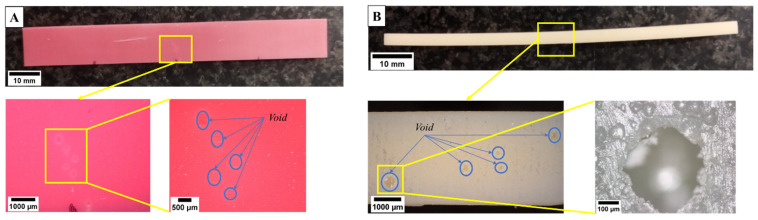
Visible voids on the printed specimen surface (LT 0.1 mm). (**A**) PLA PRO. (**B**) Dental non-castable.

Observations on the specimen fracture surfaces were also carried out for further analysis. As shown in Figure 16A,B, there are voids developed in the printed specimen during polymerization where the detailed images can be seen in the yellow rectangle. In certain cases, the existence of a void on the inside of the printed specimen is visible to the naked eye on the outer surface of the printed specimen, as shown in Figure 17. Voids on the printed part developed due to contact occurring between the printing platform and trapped air on the photopolymer resin during the polymerization process [47]. The existence of voids can reduce the mechanical properties of printed specimens [48]. The existence of voids often occurred at the bigger layer thickness, i.e., 0.1 mm. It also confirmed that the lowest flexural strength of material generally was found at LT 0.1 mm (Figure 14). Similar findings were also obtained in the previous study in which voids were often generated using bigger layer thickness [44].

**Figure 18:**
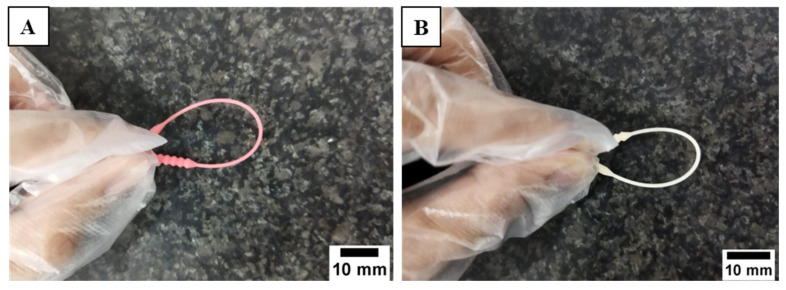
Swab neck flexibility test. (**A**) PLA PRO, (**B**) Dental non-castable.

The flexibility of the swab neck was tested to imitate the 3D-printed nasopharyngeal swab bending as it passed through the nasal cavity. A bending load on the swab neck was given by holding the swab head and bending it to the swab base section. Based on the observation that has been carried out, 3D-printed nasopharyngeal swabs were able to bend 180° without breaking, as shown in Figure 18.

### 3.4. Surface Roughness Test

**Figure 19:**
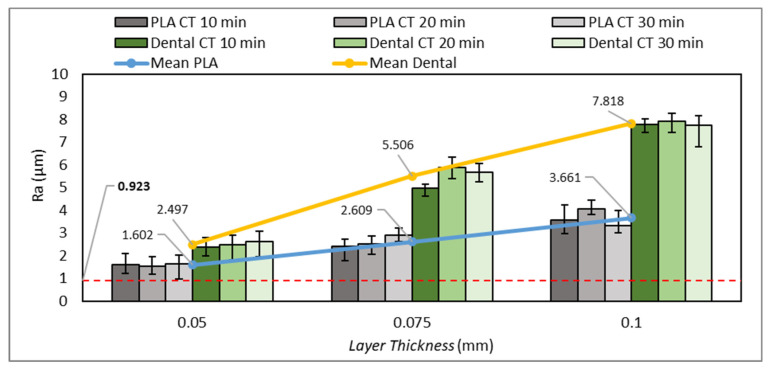
Average surface roughness value of 3D-printed nasopharyngeal swab.

Previous studies have reported that printing parameters affect the surface quality of printed parts [47,48,49]. In this section, the surface roughness test results are presented and discussed, where the influence of layer thickness and post-curing time on the surface roughness of 3D-printed nasopharyngeal swabs were also analyzed. The surface roughness test results are presented in Figure 19. The red dashed line on Figure 19 indicated the surface roughness value of the commercial flock nasopharyngeal swab. The lowest surface roughness value of the PLA PRO 3D-printed nasopharyngeal swab is 1.537 µm, obtained in the printing parameters of LT 0.05 mm at a CT of 20 min. Meanwhile, the lowest surface roughness value of dental non-castable 3D-printed nasopharyngeal swabs is 2.390 µm, which was obtained in the printing parameters of LT 0.05 mm at a CT of 10 min.

**Figure 20:**
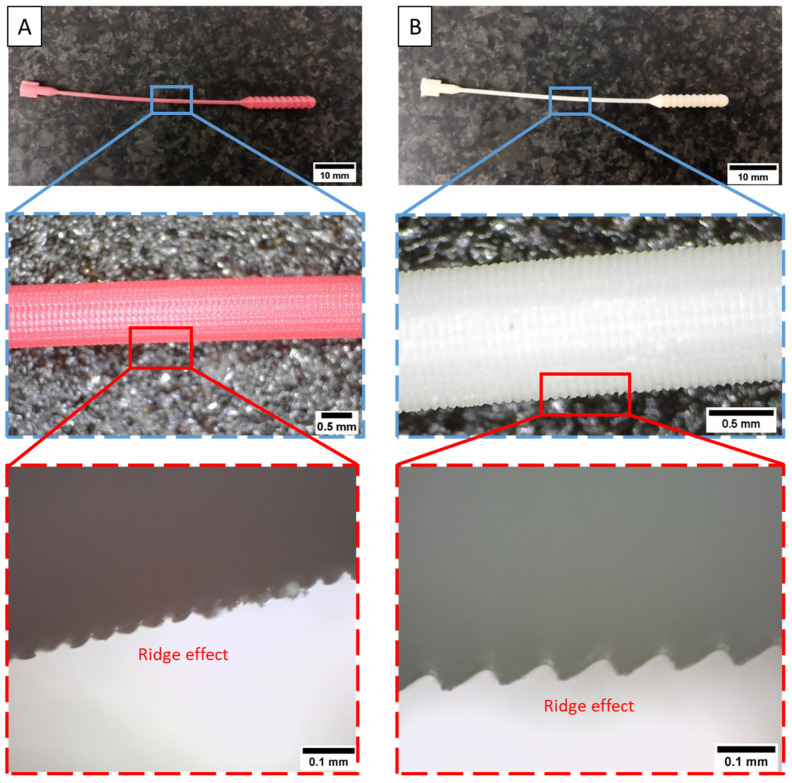
Ridge effect phenomenon on the 3D-printed nasopharyngeal swab (LT 0.1 mm). (**A**) PLA PRO, (**B**) Dental non-castable.

**Figure 21:**
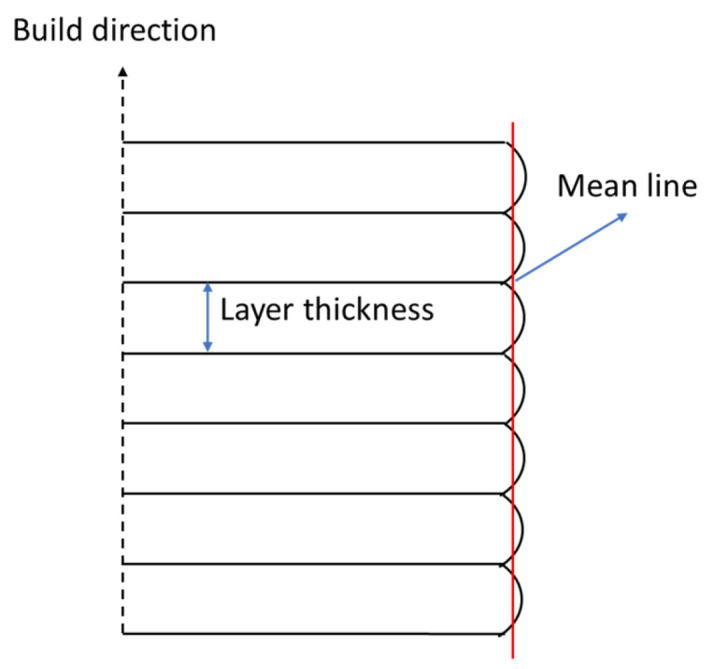
Ridge effect schematic on the printed part.

Based on Figure 19, it can be seen that the surface roughness value increases along with increasing the layer thickness size. The surface roughness of PLA PRO 3D-printed nasopharyngeal swab on CT of 10 min with an LT of 0.05 mm, 0.075 mm, and 0.1 mm are 1.625 µm, 2.412 µm, and 3.573 µm, respectively. The ANOVA result (with α = 0.05) also showed *F*-value (23.59) was higher than its *F*-crit value (3.88), implying that the surface roughness of PLA PRO was influenced by the change in LT 0.05 mm to 0.1 mm. Moreover, the ANOVA results (with α = 0.05) of CT 20 min and CT 30 min showed *F*-value (87.28) and (28.03), respectively were also far higher than *F*-crit value (3.88). Similar to PLA PRO, dental non-castable also exhibits the same phenomenon. The surface roughness of dental non-castable 3D-printed nasopharyngeal swabs on CT of 10 min with an LT of 0.05 mm, 0.075 mm, and 0.1 mm are 2.39 µm, 4.96 µm, and 7.79 µm. The ANOVA results (with α = 0.05) also showed there is a significant difference between the surface roughness of dental non-castable 3D-printed nasopharyngeal swab by changing the layer thickness size (*F*-value (411.32) > *F*-crit value (3.88)). A previous study conducted by Arnold et al. [49] and Mostafa et al. [50] confirmed this finding by reporting the surface roughness value of printed parts increases as the layer thickness size increases. This was caused by the ridge effect phenomenon on the 3D-printed nasopharyngeal swab due to the layer thickness size. The ridge effect occurs on a flat surface area, creating a small lump on each layer [51]. The lump dimension increases as the layer thickness increases. The ridge effect phenomenon on the 3D-printed nasopharyngeal swab can be seen in Figure 20. The Ridge effect cannot be removed but can be reduced by decreasing the layer thickness size [51]. The schematic of the ridge effect on the printed part is shown in Figure 21.

As shown in Figure 19, the influence of curing time on the surface roughness values of PLA PRO and dental non-castable did not show significant changes. The surface roughness value of PLA PRO 3D-printed nasopharyngeal swab on LT 0.05 mm with a CT of 10 min, 20 min, and 30 min is 1.625 µm, 1.537 µm, and 1.644 µm, respectively. The surface roughness values are relatively similar. This is also proved using the ANOVA analysis result (with α = 0.05), which shows a lower *F*-value (0.106) than its *F*-crit value (3.88), inferring that there was no significant difference between the surface roughness of 3D-printed nasopharyngeal swabs subjected to UV light post-curing treatment for 10, 20, and 30 min. Additionally, the ANOVA results on LT 0.075 mm showed *F*-value (3.09) were also lower than *F*-crit value (3.88). Similar phenomenon also happened on dental non-castable 3D-printed nasopharyngeal swab, in which the ANOVA analysis result (with α = 0.05) on LT 0.05 mm and LT 0.1 mm showed *F*-value (0.37) and (0.197), respectively were also lower than *F*-crit value (3.88). Accordingly, it can be concluded that the surface roughness of dental non-castable 3D-printed nasopharyngeal swabs was not influenced by the change in post-curing time. These results also confirm the study conducted by Sabbah et al. [52], in which UV light treatment after the printing process has no significant effect on the surface quality of the printed part. Although the ANOVA results of PLA PRO LT 0.1 mm and dental non-castable LT 0.075 mm showed significant differences in the surface roughness value by changing the curing time (*F*-value (4.05) and (12.08), respectively, were lower than *F*-crit value (3.88)), the difference in surface roughness value was slight and showed a random pattern. The surface roughness value of PLA PRO 3D-printed nasopharyngeal swab on LT 0.1 mm with a CT of 10 min, 20 min, and 30 min are 3.573 µm, 4.078 µm, and 3.333 µm, respectively, and the surface roughness value of dental non-castable 3D-printed nasopharyngeal on LT 0.075 mm with a CT of 10 min, 20 min, and 30 min are 4.697 µm, 5.883 µm, and 5.670 µm, respectively.

Based on Figure 19, the surface roughness value of the dental non-castable was higher than the PLA PRO 3D-printed nasopharyngeal swab, which means the surface quality of the PLA PRO 3D-printed nasopharyngeal swab was smoother than the dental non-castable. This finding confirmed that the difference in the chemical composition of photopolymer resin affects the surface quality of printed parts [53]. Furthermore, the lowest surface roughness value of the 3D-printed nasopharyngeal swab (0.98 µm) is close to the surface roughness value of the commercial flock nasopharyngeal swab (0.923 µm), which indicates that the 3D-printed nasopharyngeal swab has a good potential for further clinical validation in the term of surface quality.

### 3.5. Dimensional Accuracy Test

In this section, a dimensional accuracy test was performed to evaluate the influence of layer thickness and post-curing time on the dimensional accuracy of a 3D-printed nasopharyngeal swab. 3D-printed nasopharyngeal swabs were printed at every printing parameter and post-curing time treatment, then it was further measured for analysis. First, considering the dimension of a nasopharyngeal swab, this requires the swab to be printed in a 90° build orientation (alongside the Z-axis of the printing platform), which means it requires more layers than other build orientations. It has several advantages over other build orientations (e.g., 0°, 30°, 45°): (i) easy to print (no needed support), (ii) easy to remove from the printing platform, (iii) allows to print more swabs in a single batch print. More layers will increase the printing time and the possibility of failure [54].

**Figure 22:**
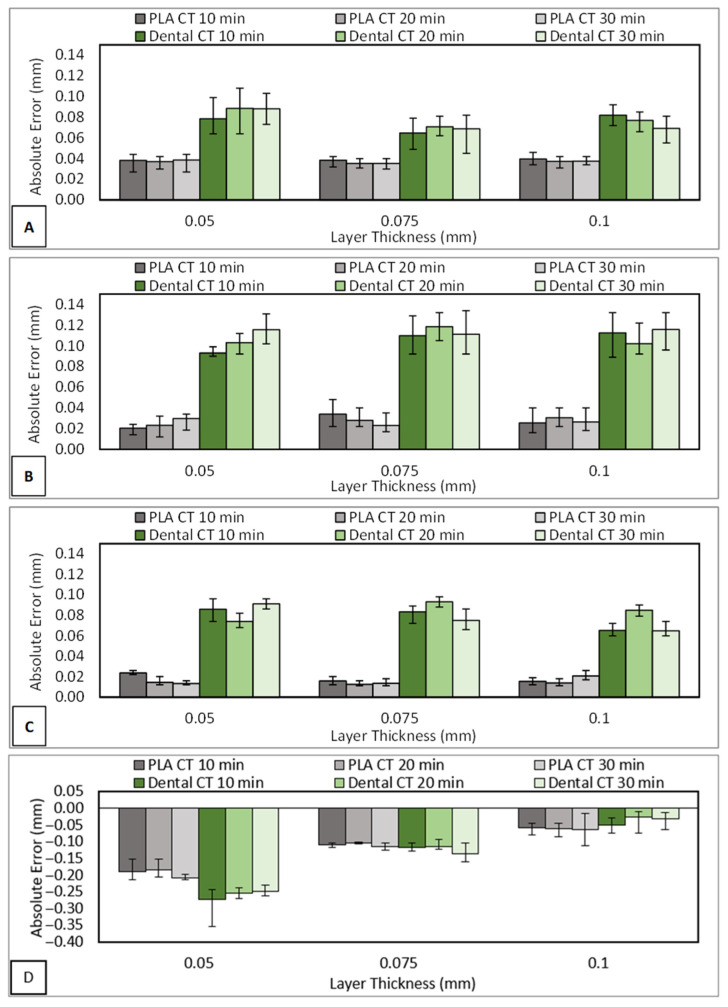
Average AE value of swab. (**A**) Neck diameter, (**B**) Head diameter, (**C**) Base diameter, (**D**) Head length.

There are four measurement dimensions of a 3D-printed nasopharyngeal swab to be measured, namely swab neck diameter (ND), swab head diameter (HD), swab head length (HL), and base diameter (BD), which they have a design dimension of 1 mm, 20.25 mm, 2.92 mm, and 2.5 mm, respectively. As shown in Figure 22, it can be seen that there are absolute errors (AE) and relative errors (RE) in positive and negative values. A positive value means that the final dimension of the 3D-printed nasopharyngeal swab is bigger than the design dimension. In contrast, a negative value means the final dimension of the 3D-printed nasopharyngeal swab is smaller than the design dimension. The greater value of AE and RE dimensions obtained either in positive or negative value means that the printing result of the nasopharyngeal swab is more inaccurate.

As shown in Figure 22A, the swab neck AE value of PLA PRO is relatively similar. The ANOVA results of PLA PRO at CT 10 min, 20 min, and CT 30 min varying with a layer thickness (with α = 0.05) showed *F*-value (0.10), (0.29), and (0.50), respectively were lower than *F*-crit value (3.88) which implied that there was no significant difference in the neck diameter AE value of PLA PRO by changing the layer thickness. Subsequently, the swab neck AE value of dental non-castable shows a fairly random pattern. In line with the PLA PRO material, dental non-castable also showed no significant influence of layer thickness on the swab neck AE value. This can be seen at CT 10 min with an LT of 0.05 mm, 0.075 mm, and 0.1 mm have an AE value of 0.078 mm, 0.065 mm, and 0.082 mm, respectively. The ANOVA analysis also reveals that there was no significant difference between the AE value of dental non-castable at CT 10 min by changing the layer thickness (with α = 0.05, *F*-value (3.165) < *F*-crit (3.88)). The ANOVA result at CT 20 min and CT 30 min (with α = 0.05) also showed a lower *F*-value (1.87) and (3.86), respectively than its *F*-crit value (3.88). Moreover, as shown in Figure 22B,C, head diameter, and base diameter have a similar phenomenon to neck diameter in terms of the influence of layer thickness on the dimensional accuracy of a 3D-printed nasopharyngeal swab. Based on this result, it can be concluded that layer thickness does not have a significant effect on the dimensional accuracy of the swab neck. Furthermore, considering the swabs were printed in a 90° build orientation (alongside the Z-axis of the printing platform), it can also be concluded that layer thickness does not have a significant effect on the dimensional accuracy measured along the X- and Y-axis of the printing platform. This finding is also contrary to the study conducted by Favero et al. [54], in which the best printing accuracy of the VP printed part was obtained with the use of the largest layer thickness.

Based on Figure 22A, CT does not significantly affect the swab neck AE value. The AE value obtained from the printing parameters varied on CT value relatively the same. This is also proved using the ANOVA analysis result (with α = 0.05) on PLA PRO at LT 0.05 mm, which showed a lower *F*-value (0.07) than its *F*-crit value (3.88), inferring that there was no significant difference between the AE value of swab neck subjected to UV light post-curing treatment for 10, 20, and 30 min. Moreover, the ANOVA result at LT 0.075 mm and LT 0.1 mm (with α = 0.05) also showed a lower *F*-value (0.81) and (0.35), respectively than its *F*-crit value (3.88). A similar phenomenon is also shown using dental non-castable material. The ANOVA results of dental non-castable at LT 0.05 mm, 0.075 mm, and 0.1 mm showed *F*-value (0.56), (0.33), and (2.98), respectively were also lower than *F*-crit value (3.88). These results confirm research conducted by McCarty et al. [55] that CT has no significant effect on the dimensional accuracy of the VP printed part.

As shown in Figure 22D, the head length dimension generally shows negative values, indicating that the final dimension of the head length was smaller than the design dimension. This may be due to the 3D-printed nasopharyngeal swabs being printed in a 90° build orientation (alongside the Z-axis of the printing platform), which can be associated with the layer numbers to be cured in the printing process. The layer number of 3D-printed nasopharyngeal swabs with an LT of 0.1 mm, 0.075 mm, and 0.05 mm are 884, 1179, and 1769, respectively. LT 0.05 mm has the highest AE deviation value, followed by LT 0.075 mm and LT 0.1 mm. Furthermore, the ANOVA results (with α = 0.05) on PLA PRO at CT 10 min, 20 min, and CT 30 min showed *F*-value (76.24), (66.49), and (52.13), respectively, were far higher than *F*-crit value (3.88). Accordingly, it can be concluded that the head length AE value was influenced by the change in layer thickness. A similar pattern is also shown using dental non-castable material. The smallest AE value was obtained using the largest layer thickness. The greater the number of layers that need to be cured in the printing process, the greater the possibility for error, which could result in decreased dimensional accuracy of the printed part [54]. This result confirms the study conducted by Boca et al. [56] and Favero et al. [54], in which the best printing accuracy of VP was obtained on the largest layer thickness. In addition, it also can be mentioned that layer thickness also has a significant effect on the dimensional accuracy measured along the Z-axis of the printing platform. This finding also can be concluded that decreasing the layer thickness does not necessarily increase the dimensional accuracy of the printed part. Moreover, these results also showed that the error in length was greater than the error in diameter. Based on the data presented in Figure 22, the highest deviation of AE value on PLA PRO and dental non-castable are −0.21 mm and −0.274 mm, respectively, which were obtained in the printing parameters of ET 4 s LT 0.05 mm. Subsequently, the smallest deviation of AE value on PLA PRO and dental non-castable are 0.012 mm and 0.065 mm, respectively, which were obtained in the printing parameters of ET 4 s LT 0.075 mm. The smallest AE value obtained in the present study, included in the criterion limit recommendation for clinical use, is 0.25 mm [55].

### 3.6. Sample Collection

**Figure 23:**
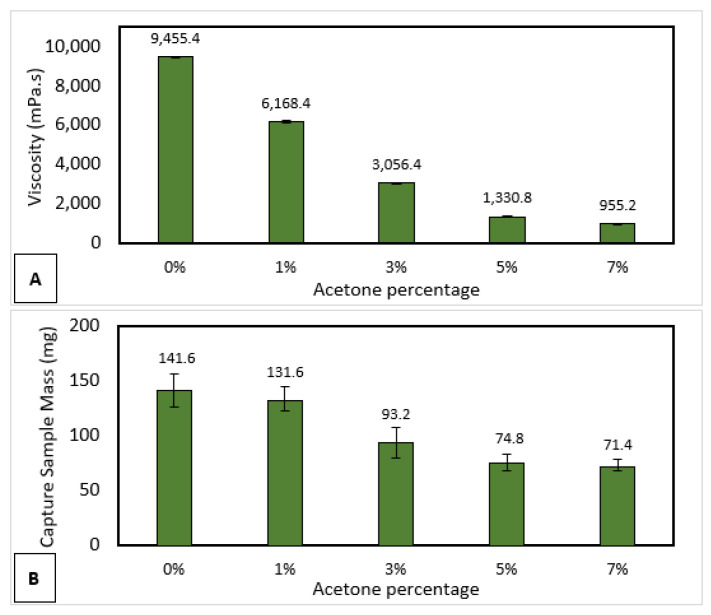
(**A**) Viscosity measurement of artificial mucus with a different concentration of epoxy resin bisphenol A and acetone. (**B**) Sample collection test result.

A sample collection test was carried out to measure the ability of a 3D-printed nasopharyngeal swab’s ability to carry several samples coming out from the nasal cavity. A mixture of epoxy resin bisphenol A and acetone was used to create artificial mucus liquid to represent human mucus. Human mucus has varying viscosity values depending on many factors, including patients with respiratory tract diseases such as rhinitis, sinusitis, and bronchitis [57]. Human mucus viscosity of patients with chronic bronchitis is around 1–80 Pa.s, then in certain diseases such as bronchorrhea, human mucus viscosity shows a low value below 5 Pa.s [58]. Artificial mucus with various viscosity can be achieved by changing the percentage of acetone, as shown in Figure 23A. There was a decrease in viscosity as the percentage of acetone increased. The result showed that the highest artificial mucus viscosity was 9455.4 mPa.s, whereas the lowest artificial mucus viscosity was 955.2 mPa.s. As shown in Figure 23B, the proposed swab head design was able to carry an artificial mucus sample of 141.6 mg at a viscosity of 9455.4 mPa.s. The proposed head swab design demonstrated an increase in sampling ability as an increase in the viscosity of artificial mucus.

### 3.7. Manufacturing Process and Cost Analysis

Previous studies reported that the printing parameters have an influence on the mechanical properties [35,41,57,59], dimensional accuracy [52,53,54], and surface roughness of printed parts [47,48,49]. The selection of printing parameters in the swab fabrication via VP is very important to ensure the 3D-printed nasopharyngeal swab is suitable for use before being clinically tested. As shown in Table 6, the printing parameter, especially layer thickness, affects the printing time of a 3D-printed nasopharyngeal swab. Accordingly, the proper selection of printing parameters will provide effectiveness in production time without compromising the quality of the 3D-printed nasopharyngeal swab. A series of in-depth explanations have been presented in the previous section related to the influence of layer thickness and post-curing time on the mechanical properties, surface roughness, and dimensional accuracy of 3D-printed nasopharyngeal swabs. Hence, this section will provide the manufacturing process and cost analysis to complete the explanation in the previous section.

**Figure 24:**
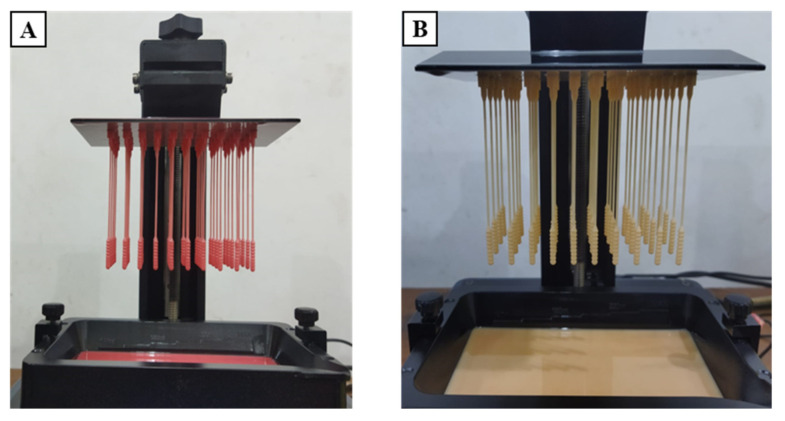
3D-printed nasopharyngeal final prints. (**A**) PLA PRO, (**B**) Dental non-castable.

Considering the printing volume of Anycubic Photon Ultra, it can be able to print 3D-printed nasopharyngeal swabs of 50 pieces per batch, as shown in Figure 24, which takes an estimated 10.5 mL or about 0.21 mL per swab of photopolymer resin to print. Even the printing capacity could increase to 100 pieces of 3D-printed nasopharyngeal swabs per batch by providing a closer distance between the swabs. As shown in Table 6, the printing time of PLA PRO 3D-printed nasopharyngeal swabs with an LT of 0.05 mm, 0.075 mm, and 0.1 mm are 7 h 48 min, 5 h 12 min, and 3 h 54 min, respectively. Whereas the printing time of dental non-castable 3D-printed nasopharyngeal swabs with an LT of 0.05 mm, 0.075 mm, and 0.1 mm are 10 h 1 min, 6 h 40 min, and 5 h 2 min, respectively. As described in the previous section, every layer thickness value has advantages and disadvantages. Smaller layer thickness has better mechanical properties and surface roughness. However, it has a longer printing time, whereas bigger layer thickness has better dimensional accuracy in length but has a shorter printing time. Ultimately, the authors suggest using an LT of 0.05 mm to print a 3D-printed nasopharyngeal swab. Assuming the swab fabrication using PLA PRO photopolymer resin, LT of 0.05 mm and 100 pieces were printed per batch, for the complete manufacturing process from calibration to post-processing, the swab is estimated to take about 8.5 h. Hence, the production capacity of a 3D-printed nasopharyngeal swab is about 5 min/swab/machine.

**Figure 25:**
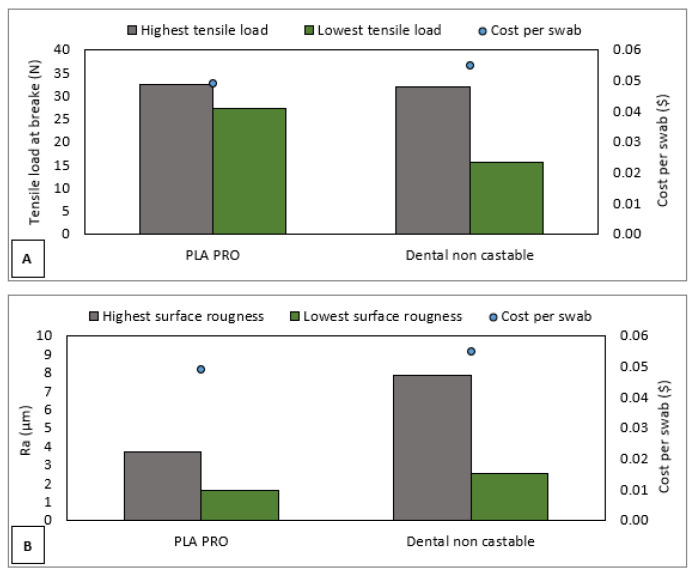
Cost comparison to the swab characteristics. (**A**) Tensile load at the break, (**B**) Surface roughness.

eSun PLA PRO and Anycubic dental non-castable photopolymer resin were chosen as source materials to fabricate a 3D-printed nasopharyngeal swab. Alcohol liquid was also used to wash the swab after the printing process finished. Furthermore, additional materials such as tissue, paper funnel filter, and FEP film replacement were also used. The price to obtain Anycubic Photon Ultra 3D printer (Shenzhen, China) and Anycubic Wash and Cure 2.0 (Shenzhen, China) were around USD600 and USD228, respectively. Based on the marketplace, the cost of 500 mL of eSun PLA PRO, 500 mL of Anycubic dental non-castable, and 5 L of alcohol liquid are about USD23.3, USD50, and USD10, respectively. Further cost analysis found that the cost incurred to fabricate PLA PRO swabs is about USD0.049 per swab, whereas the cost of dental non-castable swabs is about USD0.055 per swab. As shown in Figure 25, considering the mechanical properties, surface roughness, and fabrication cost of a 3D-printed nasopharyngeal swab, it should be noted that a PLA PRO swab is less expensive than a dental non-castable swab.

## 4. Conclusions

The manufacturing process, characteristics, performance, defectiveness, and influence of printing parameters and post-curing process of nasopharyngeal swabs using vat photopolymerization 3D-printing have also been evaluated. A multi-part component 3D-printed nasopharyngeal swab was proposed, in which the swab and handle were manufactured separately; thus, the swab size became shorter. The shorter swab size can reduce the printing time and photopolymer resin material. In addition, the proposed swab design also gives stress concentration or breaking point at a particular position to ensure the patient’s safety. 3D-printed nasopharyngeal swabs show outperforms in tensile testing compared to the commercial flock nasopharyngeal swabs. The flexural test results showed that PLA PRO has greater flexural strength than dental non-castable. However, the swab neck flexibility test showed that both PLA and dental non-castable 3D-printed nasopharyngeal swabs were able to bend 180°. Subsequently, the PLA PRO swab showed a lower surface roughness value than the dental non-castable swab, indicating that the PLA PRO 3D-printed nasopharyngeal swab was smoother than the dental non-castable. Artificial mucus with various viscosity was set to measure the sample collection ability of a 3D-printed nasopharyngeal swab. Both PLA PRO and dental non-castable photopolymer resin have shown engaging material as source material for swab fabrication. It should be noted that 3D-printed nasopharyngeal swab needs to be clinically tested before being widely applied. The selection of printing parameters and post-curing time treatment affected the characteristics of 3D-printed nasopharyngeal swabs. Smaller layer thickness and longer curing time will create a greater performance in tensile testing of a nasopharyngeal swab. In addition, smaller layer thickness provides a better surface quality but lacks dimensional length accuracy. Lastly, rapid prototyping and fabrication via VAT photopolymerization 3D-printing is an adorable option to consider for nasopharyngeal swab production, especially in a critical situation. 3D printing allows multiple designs to be printed, tested, and then quickly redesigned. Moreover, various types of materials and printing processes are available, which means that there are many alternatives for producing nasopharyngeal swabs using 3D printing technology.

## Figures and Tables

**Table 1:** Photopolymer resin chemical composition.

eSun PLA PRO		
Chemical Name	CAS No.	% by Weight
Acrylates aliphatic urethane	68987-79-1	40–50%
Monomer	13048-33-4	20–40%
Photoinitiators	75980-60-8	3–5%
Color pigment		2–5%
Anycubic dental non-castable		
Chemical Name	CAS No.	% by weight
Epoxy acrylate resin	61788-97-4	40–50%
Monomer	13048-33-4	20–40%
Photoinitiators	947-19-3	3–5%
Color pigment		2–5%
eSun standard		
Chemical Name	CAS No.	% by weight
Urethane acrylate	68987-79-1	35%
Trimethylolpropane triacrylate	15625-89-5	25%
Tripropyleneglycol diacrylate	42978-66-5	19.5%
2-Phenoxyethyl acrylate	48145-04-6	15%
Trimethylbenzoyldiphenylphosphine oxide	75980-60-8	3%
Phenylbis phosphine oxide	162881-26-7	1%
BYK 1790	128-37-0	1%
UV Color pigment	133-86-4/482-89-3	0.5%

**Table 2:** Photopolymer resin properties.

eSun PLA PRO		
Properties	Value	Unit
Density	1.09–1.10	g/cm^3^
Elongation at break	25–28	%
Flexural strength	36–49	MPa
Hardness	78–80	Shore D
IZOD impact strength	32–36	J/m
Tensile strength	37–48	MPa
Viscosity	200–300	mPa.s
Anycubic dental non-castable		
Properties	Value	Unit
Density	1.05–1.13	g/cm^3^
Elongation at break	11–20	%
Hardness	88	Shore D
Shrinkage	1.86–2.12	%
Tensile strength	42–62	Mpa
Viscosity	100–150	mPa.s
eSun standard		
Properties	Value	Unit
Density	1.08–1.13	g/cm^3^
Elongation at break	28–36	%
Flexural strength	46–72	Mpa
Hardness	78–82	Shore D
IZOD impact strength	14–42	J/m
Tensile strength	46–67	Mpa
Viscosity	170–200	mPa.s

**Table 3:** Printing parameters for 3D-printed nasopharyngeal swabs and flexural test specimens.

Slicing Setting	
Parameter	Value
Layer Thickness (mm)	0.05, 0.075, 0.1
Normal Exposure Time (s)	4
Off Time (s)	1
Bottom Exposure Time (s)	40
Bottom Layers	4
Z Lift Distance (mm)	4, 5
Z Lift Speed (mm/s)	1, 2
Z Retract Speed (mm/s)	1, 2
Anti-alias	4
Build orientation (deg)	90
Post-processing	
Washing time (minute)	5
Curing time (minute)	10, 20, 30

**Table 4:** Anycubic Photon Ultra 3D printer specifications.

Printing	
System	Anycubic Photon Ultra
Software	Anycubic photon workshop
Operation	2.8-inch color TFT screen
Connectivity	USB memory stick
Specifications	
Technique	Digital Light Projection
Light source	UV-LED (wavelength 405 nm)
XY resolution	0.08 mm 1280 × 720 (720 p)
Z axis accuracy	0.01 mm
Suggested layer thickness	0.01–0.15 mm
Suggested print speed	Max 50 mm/h
Rated power	15 W
Physical Dimensions	
Dimensions	222 × 227 × 383 mm (L × W × H)
Build volume	102.4 × 57.6 × 165 mm (L × W × H)
Materials	405 nm UV resin
Net weight	~4 kg

**Table 5:** Anycubic Wash and Cure 2.0 technical specifications.

Specification	
Control method	Digital displays + LED dual light, knob
Rated power	25 W
Input voltage	AC 110/220 V 50/60 Hz
UV LED	405 nm
Time mode	1~60 min
Physical Dimensions	
Machine size	225 × 235 × 365 mm (L × W × H)
Maximum wash volume	120 × 74 × 165 mm (L × W × H)
Maximum curing volume	140 × 165 mm (D × H)
Net weight	~3.7 kg

**Table 6:** Design of experiment.

Material	No	Layer Thickness (mm)	Normal Exposure Time (s)	Z Speed (mm/s)	Printing Time		Post-Curing Time (Menit)
					3D-Printed Nasopharyngeal Swab	Flexural Test Specimen	
PLA PRO	1	0.05	4	2	7 h 48 min	1 h 8 min	10
	2	0.05	4	2	7 h 48 min	1 h 8 min	20
	3	0.05	4	2	7 h 48 min	1 h 8 min	30
	4	0.075	4	2	5 h 12 min	46 min	10
	5	0.075	4	2	5 h 12 min	46 min	20
	6	0.075	4	2	5 h 12 min	46 min	30
	7	0.1	4	2	3 h 54 min	35 min	10
	8	0.1	4	2	3 h 54 min	35 min	20
	9	0.1	4	2	3 h 54 min	35 min	30
Dental Non-castable	10	0.05	4	1	10 h 1 min	1 h 41 min	10
	11	0.05	4	1	10 h 1 min	1 h 41 min	20
	12	0.05	4	1	10 h 1 min	1 h 41 min	30
	13	0.075	4	1	6 h 40 min	1 h 7 min	10
	14	0.075	4	1	6 h 40 min	1 h 7 min	20
	15	0.075	4	1	6 h 40 min	1 h 7 min	30
	16	0.1	4	1	5 h 2 min	51 min	10
	17	0.1	4	1	5 h 2 min	51 min	20
	18	0.1	4	1	5 h 2 min	51 min	30

## Data Availability

The data related to the work are available upon request.
